# Stress reduction through cortical bone thickening improves bone mechanical behavior in adult female Beclin-1^+/−^ mice

**DOI:** 10.3389/fbioe.2024.1357686

**Published:** 2024-03-27

**Authors:** Jiaojiao Yang, Qilin Pei, Xingfan Wu, Xin Dai, Xi Li, Jun Pan, Bin Wang

**Affiliations:** ^1^ Key Laboratory for Biorheological Science and Technology of Ministry of Education, College of Bioengineering, Chongqing University, Chongqing, China; ^2^ Institute of Life Sciences, College of Basic Medicine, Chongqing Medical University, Chongqing, China; ^3^ Department of Biomedical Engineering, Fourth Military Medical University, Xi’an, China

**Keywords:** Beclin-1, bone strength, mechanical property, finite element model, cortical bone, adult female, stress, safety factor

## Abstract

Fragility fractures, which are more prevalent in women, may be significantly influenced by autophagy due to altered bone turnover. As an essential mediator of autophagy, Beclin-1 modulates bone homeostasis by regulating osteoclast and chondrocyte differentiation, however, the alteration in the local bone mechanical environment in female Beclin-1^+/−^ mice remains unclear. In this study, our aim is to investigate the biomechanical behavior of femurs from seven-month-old female wild-type (WT) and Beclin-1^+/−^ mice under peak physiological load, using finite element analysis on micro-CT images. Micro-CT imaging analyses revealed femoral cortical thickening in Beclin-1^+/−^ female mice compared to WT. Three-point bending test demonstrated a 63.94% increase in whole-bone strength and a 61.18% increase in stiffness for female Beclin-1^+/−^ murine femurs, indicating improved biomechanical integrity. After conducting finite element analysis, Beclin-1^+/−^ mice exhibited a 26.99% reduction in von Mises stress and a 31.62% reduction in maximum principal strain in the femoral midshaft, as well as a 36.64% decrease of von Mises stress in the distal femurs, compared to WT mice. Subsequently, the strength-safety factor was determined using an empirical formula, revealing that Beclin-1^+/−^ mice exhibited significantly higher minimum safety factors in both the midshaft and distal regions compared to WT mice. In summary, considering the increased response of bone adaptation to mechanical loading in female Beclin-1^+/−^ mice, our findings indicate that increasing cortical bone thickness significantly improves bone biomechanical behavior by effectively reducing stress and strain within the femoral shaft.

## 1 Introduction

Fragility fractures, characterized by compromised bone strength and increased bone fragility, primarily arise from age-related bone loss or inadequate peak bone mass at maturity (Ensrud et al., 1995; Melton, 1997; Clynes et al., 2020). Fractures occur twice as frequently in women as in men, with women accounting for 75% of hip fractures (Jordan and Cooper, 2002). Globally, osteoporosis affects approximately 200 million women, with a prevalence of one in three women over the age of 50 experiencing an osteoporotic fracture (de Villiers and Goldstein, 2022). The biomechanical response of bones to stress begins to decrease after maturity. Most of the changes were observed in biomechanical compared to architectural properties and female bones are more severely affected by aging (Mumtaz et al., 2020). The correlations between trabecular bone microstructure and site-specific as well as age-related factors have previously been shown to exhibit greater prominence in females (Glatt et al., 2007; Lochmuller et al., 2008). Age-related reductions in cortical bone thickness and cross-sectional area were observed in elder females, suggesting a relationship between the risk of fracture and morphological changes in cortical bone (Imamura et al., 2019). Considerable efforts focus on mitigating age-related bone loss postmenopause, while comparatively less on assessing peak skeletal mass and fracture risk among nonpregnant adult women (Rogers et al., 2000; Mazzuoli et al., 2002; Boschitsch et al., 2017). The impact of fragility fractures may be underestimated in adult females, who are uniquely affected by gender without the confounding influence of age, hormones, or other factors.

Autophagy is highly involved in bone metabolism, acts as a primary determinant of bone mass, structure, and functional remodeling (Wang et al., 2023). All types of bone cells demonstrated a basal level of autophagic activity (Arai et al., 2019; Yin et al., 2019). Autophagy preserves metabolic energy homeostasis and regulates mineralization and absorption, playing a crucial role in bone regeneration (Mizushima et al., 2008; Yin et al., 2019; Wang et al., 2023). Our previous study found that mechanical stimulation within the physiological range induces protective autophagy in osteocytes (Zhang et al., 2018). Impaired autophagy in osteoblasts triggers endoplasmic reticulum stress and results in significant bone loss (Li et al., 2018). Pathological dysregulation of autophagy initiates the onset and development of osteoporosis. Aging, estrogen deficiency, and glucocorticoids induce downregulation in autophagic activity, thereby contributing to the development of osteoporosis (Yin et al., 2019). Estrogen has been confirmed to enhance the survival and functionality of human osteoblasts by promoting autophagy (Gavali et al., 2019). Furthermore, autophagy could preserve the functionality of bone marrow mesenchymal stem cells, preventing bone loss caused by the lack of estrogen. Reduced autophagy in osteoblasts of female mice, associated with increased oxidative stress, may contribute to osteoporosis development, suggesting that autophagy could represent a new therapeutic target for ameliorating osteoporosis in women (Camuzard et al., 2016). As a key regulator of autophagy, Beclin-1 has been confirmed to modulate bone homeostasis by regulating osteoclast and chondrocyte differentiation; A deficiency of Beclin-1 in osteoclasts leads to a decrease in cancellous bone mass and an increase in cortical bone thickness in mice, accompanied by impaired chondrocyte differentiation (Arai et al., 2019). Furthermore, the expression of Beclin-1 decreases in both human osteoarthritis patients and ovariectomized osteoporotic mice (Qi et al., 2017; Zhang et al., 2019). Despite these insights into the molecular mechanisms of Beclin-1, the impact on factors related to bone fragility such as whole-bone morphology, tissue horizontal strain, and bone mechanical behavior remains unexplored.

Finite element (FE) models of femurs based on quantitative computed tomography (QCT) have been extensively used to estimate bone stiffness and strength (Mann et al., 2008; Benca et al., 2017; Mosleh et al., 2020; Sullivan et al., 2020; Varga et al., 2020), which has been applied to predict fracture risk in bone diseases and metastases (Arrington et al., 2006; Anez-Bustillos et al., 2014; Celik et al., 2019; Falcinelli et al., 2019; Nandi et al., 2022; Verbruggen and McNamara, 2023). Theories of bone adaptation that have been developed to predict changes in bone shape and density are based on strain (Patel et al., 2014; Yang et al., 2014; Razi et al., 2015; Yang et al., 2017; Javaheri et al., 2020; Katz and Yosibash, 2020), stresses (San Antonio et al., 2012; Celik et al., 2019; Falcinelli et al., 2019; Bruce Ralphin Rose, 2020), and strain energy density (SED) (Cresswell et al., 2018; Lu et al., 2019). The objective of this study was to characterize changes in the biomechanical environment of the femurs of female Beclin-1^+/−^ mice compared to those of female WT mice by FE analysis based on micro-computed tomography (Micro-CT) images. The observed changes in the whole bone mechanical behaviors in female Beclin-1^+/−^ mice may provide information on the prediction of fracture associated with cortical bone and help to elucidate the mechanisms of fracture.

## 2 Methods

### 2.1 Animal model

All animal experiments carried out with the approval of the Institutional Animal Care and Use Committee (IACUC) of Chongqing Medical University. Beclin-1^+/−^ mice with a C57BL/6J background were obtained from Beth Levine’s laboratory (Qu et al., 2003), and the age- and gender-matched wild-type (WT) C57BL/6 mice served as a control. In this study, seven-month-old female Beclin-1^+/−^ mice (*n* = 5) and WT mice (*n* = 5), which were individually housed in ventilated cages under standard laboratory conditions with access to ample food and water (22°C, 12-h light/dark cycle), were used. Mice were euthanized by cervical dislocation according to the AVMA Guidelines (American Veterinary Medical Association, 2020), the femurs were then harvested, cleaned of soft/adherent tissues, and fixed in 4% paraformaldehyde for 48 h. The right femurs were subjected to micro-CT analysis to examine bone architecture followed by three-point bending tests to determine bone mechanical properties.

### 2.2 Micro-CT imaging and analysis

The intact femurs were scanned by micro-CT at an isotropic voxel size of 10.5 μm (μCT 40, Scanco Medical AG; 70 kVp, 0.5 mm Al filter). Quantitative parameters related to the three-dimensional microstructures of the cancellous bone of the distal metaphyseal femoral bone and the cortical bone of the midshaft femoral bone were recorded according to previously published guidelines (Bouxsein et al., 2010). The phenotypic parameters for the cortical bone included total cross-sectional area inside the periosteal envelope (Tt.Ar, mm^2^), the area of cortical bone (Ct.Ar, mm^2^), Ct. Ar/Tt.Ar (%), cortical thickness (Ct.Th, mm), endocortical perimeter (Ec.Pm, mm), periosteal perimeter (Ps.Pm, mm), polar moment of inertia (pMOI, mm^4^), minimum moment of inertia (*I*_min_, mm^4^), and cortical tissue mineral density (Ct.TMD, mg HA/cm^3^). The phenotypic parameters for the cancellous bone included the bone volume fraction (BV/TV, %), trabecular thickness (Tb.Th, mm), trabecular number (Tb.N, 1/mm), trabecular separation (Tb.Sp, mm), structure model index (SMI, SMI will be 0 for parallel plates and three for cylindrical rods, and four for perfect spheres), and trabecular bone mineral density (vBMD, mg HA/cm^3^).

### 2.3 Biomechanical testing

After micro-CT scanning, the femurs were subjected to three-point bending until failure to assess their mechanical properties. The femur was loaded with a span of 7 mm at a deflection rate of 0.05 mm/s using a univert mechanical test system (Cellscale, Waterloo. Ontario, Canada). Force-displacement data were collected every 0.2 s, recorded to generate the load-displacement curve; subsequentially, we calculated the maximum force (*F*, N), stiffness (*K*, N/mm), post-yield displacement (PYD, mm), work-to-fracture (Nmm), elastic modulus (*E*, GPa) following established guidelines (Jepsen et al., 2015). Eq. 1 were used to calculate the elastic modulus (*E*):

$$E=\frac{{KL}^{3}}{48I_{\min}}$$ (1)

where *K* is the stiffness, *L* is the loading span, *I*_min_ is the minimum moment of inertia.

### 2.4 Finite element analysis

**FIGURE 1:**
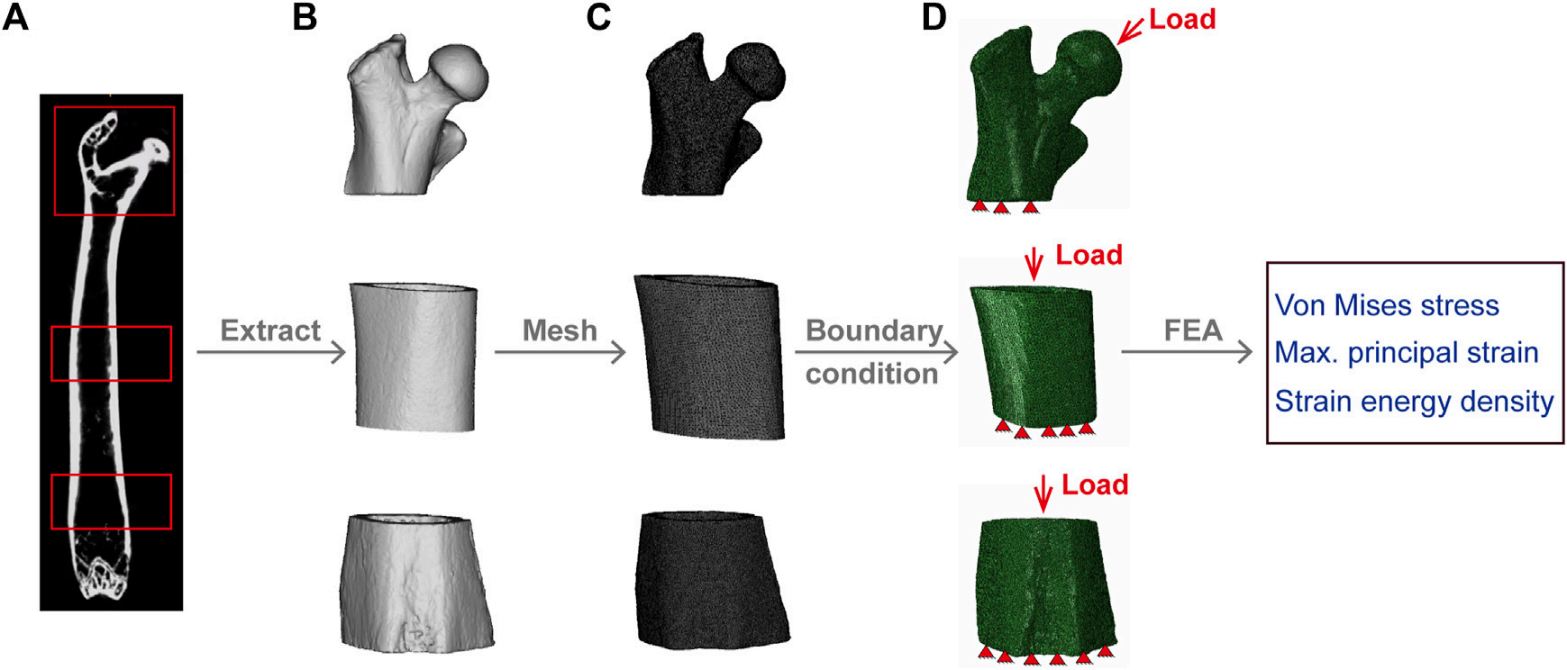
Finite Element model development. **(A)** Micro-CT image of mouse femur. **(B)** Segmentation and reconstruction of micro-CT images to generate proximal, midshaft, and distal femur models. **(C)** Model extraction of proximal, midshaft femoral and distal femur model with tetrahedral elements. **(D)** Application of load and boundary conditions; red triangles represent the distal femur fixed in all directions. The load on the proximal femur is uniformly distributed on the surface of the femoral head, while for midshaft and distal region, is coupled to the cortical bone surface of the proximal femur.

DICOM files obtained from the micro-CT scans of the femurs of the WT and Beclin-1^+/−^ mice were imported into the MIMICS software (19.0, Materialise, Belgium) for segmentation and reconstruction to generate 3D models of the proximal femur, midshaft femur, and distal femur (Figure 1B). The volumes of interest (VOIs) for the proximal femur were defined as spanning from the most proximal point of the femoral head to 4 mm in the distal direction, while the VOIs for the distal femur were defined as spanning 2 mm toward the midshaft from the distal growth plate (Figure 1A). Each model was meshed to produce a three-dimensional FE model consisting of four-node tetrahedral elements (Figure 1C).

Bone heterogeneous material properties, assumed to be linear elastic, were mapped to create structural anisotropy by varying the distribution of density (Verbruggen and McNamara, 2023). In the process of determining the femur’s material properties, the bone ash density (*ρ*_ash_, g/cm^3^) was calculated from the Hounsfield units (HU) (Les et al., 1994; Dragomir-Daescu et al., 2010) based on CT-derived bone mineral density (*ρ*_QCT_) by Eq. 2 and used to obtain the elastic modulus (*E*, GPa) according to Eq. 3 (Keller, 1994; Knowles et al., 2016; Lu et al., 2019). A Poisson ratio of 0.3 was assigned to all models (Webster et al., 2008).

$$\rho_{ash}=0.04162+0.000854\text{HU}$$ (2)

$$E=10.5\rho_{ash}^{2.09}$$ (3)

To reflect the peak physiological loading on murine long bone, apply forces in the proximal-distal direction equal to 120% of the mouse’s weight and 10.9% in the anterior-posterior direction (Charles et al., 2018); and the distal surfaces were fixed in all directions (Figure 1D). The load for the proximal femur was evenly distributed on a small circular surface of the femoral head, coupled to a reference node located at the center of the mass of the femoral head (Blanchard et al., 2013), and the surface area of the proximal cortical bone was coupled to a reference point perpendicular to the center of the surface area for the midshaft and distal regions (Verbruggen and McNamara, 2023). Linear elastic FE analysis was performed in Abaqus (Dassault Systèmes, version 2022). The von Mises stress, maximum principal strain, and SED were quantitatively evaluated for each region as parameters describing the mechanical environment of the mouse femurs.

To predict the fracture risk, the minimum safety factor (SF) defined as given in Eq. 4 was calculated for each model (Taddei et al., 2006), where the ultimate strength (*S*, MPa) of the bone tissue was obtained from the apparent ash density by Eq. 5 (Keller, 1994; Fleps et al., 2020) and the maximum von Mises stress (*σ*, MPa) was computed from the FE model.

$$SF=\frac{S}{\sigma }$$ (4)

$$S=116\rho_{ash}^{2.03}$$ (5)

### 2.5 Data analysis

The differences in the femoral morphology, tissue-level stress and strain, and whole-bone mechanical properties between WT and Beclin-1^+/−^ mice were assessed using unpaired *t*-tests with the GraphPad Prism software (GraphPad Software, USA) to determine the effects of Beclin-1 deficiency on these outcome parameters. Linear regression analysis with standard errors was employed to examine associations between bone morphological parameters and bone mechanical parameters, as well as between bone morphological parameters and biomechanical indicators predicted by the FE model for the femurs. Pearson correlation analysis was used to evaluate all relevant correlations. The results are presented as the mean ± standard deviation, with statistical significance defined as a value of *p* < 0.05.

## 3 Results

### 3.1 Beclin-1 deficiency increased cortical bone mass (not trabecular bone) in female mice

**FIGURE 2:**
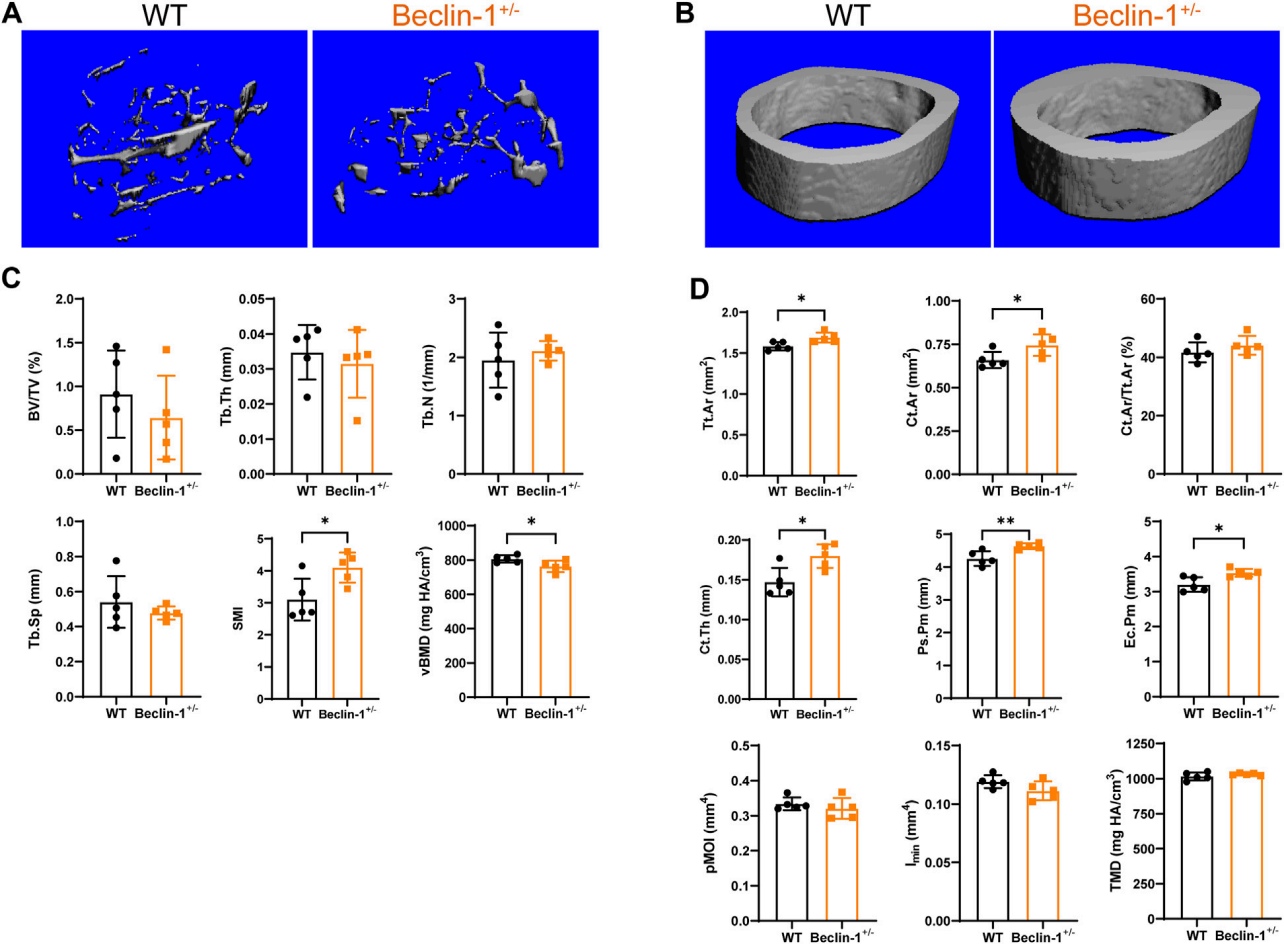
Altered cortical bone microstructure in Beclin-1^+/−^ female mice. Representative Micro-CT reconstruction images of **(A)** trabecular bone and **(B)** cortical bone of female WT and Beclin-1^+/−^ mice. Quantitative analyses of femur’s trabecular bone **(C)** and cortical bone **(D)**. Significant difference: *p < 0.05.

Compared to WT mice, Beclin-1^+/−^ mice exhibited a reduction in body weight (CTL: 25.70 ± 1.16 g, Beclin-1^+/−^: 23.12 ± 0.6 g, *p* < 0.05, *n* = 5 per group), significant reduced trabecular bone mineral density, notable increased SMI, but a slight decrease in trabecular bone mass (BV/TV), thickness, and space along with minor increment of trabecular number (Figures 2A, C). The Tissue mineral density in cortical bone did not exhibit significant changes. Significant enhancements were observed in cortical area and cortical thickness, and cortical bone perimeters (both Ps.Pm and Ec.Pm), as well as Tt. Ar; while there were no significant changes in Cortical area fraction (Ct.Ar/Tt.Ar), the minimum and polar moments of inertia at the cortical midshaft regions (Figures 2B, D).

### 3.2 Beclin-1 deficiency enhanced whole-bone mechanical properties in female mice

The three-point bending results (Table 1) showed that Beclin-1^+/−^ mice exhibited significantly increased maximum force with 63.94% difference and stiffness with 61.18% difference, while a reduction of PYD (−35.0% difference) and similar work-to-fracture, compared to WT mice. Moreover, the elastic modulus was 72.3% greater for the femurs from the Beclin-1^+/−^ mice.

**TABLE 1:** Comparison of femur bone mechanical properties between 7-month-old female WT and Beclin-1^+/−^ mice.

	WT	Beclin-1^+/−^	Diff (%)
Max Force (N)	10.40 ± 0.67	17.05 ± 1.97**	63.94
Stiffness (N/mm)	63.80 ± 11.98	102.83 ± 7.84**	61.18
PYD (mm)	0.15 ± 0.01	0.10 ± 0.03*	−35.0
Work-to-fracture (Nmm)	2.02 ± 1.15	2.32 ± 0.70	14.85
Elastic modulus (GPa)	3.83 ± 0.75	6.61 ± 0.38**	72.30

Differences are calculated as (Beclin-1^+/−^WT)/WT×100. Significant difference: *p < 0.05, **p < 0.001 relative to WT.

**FIGURE 3:**
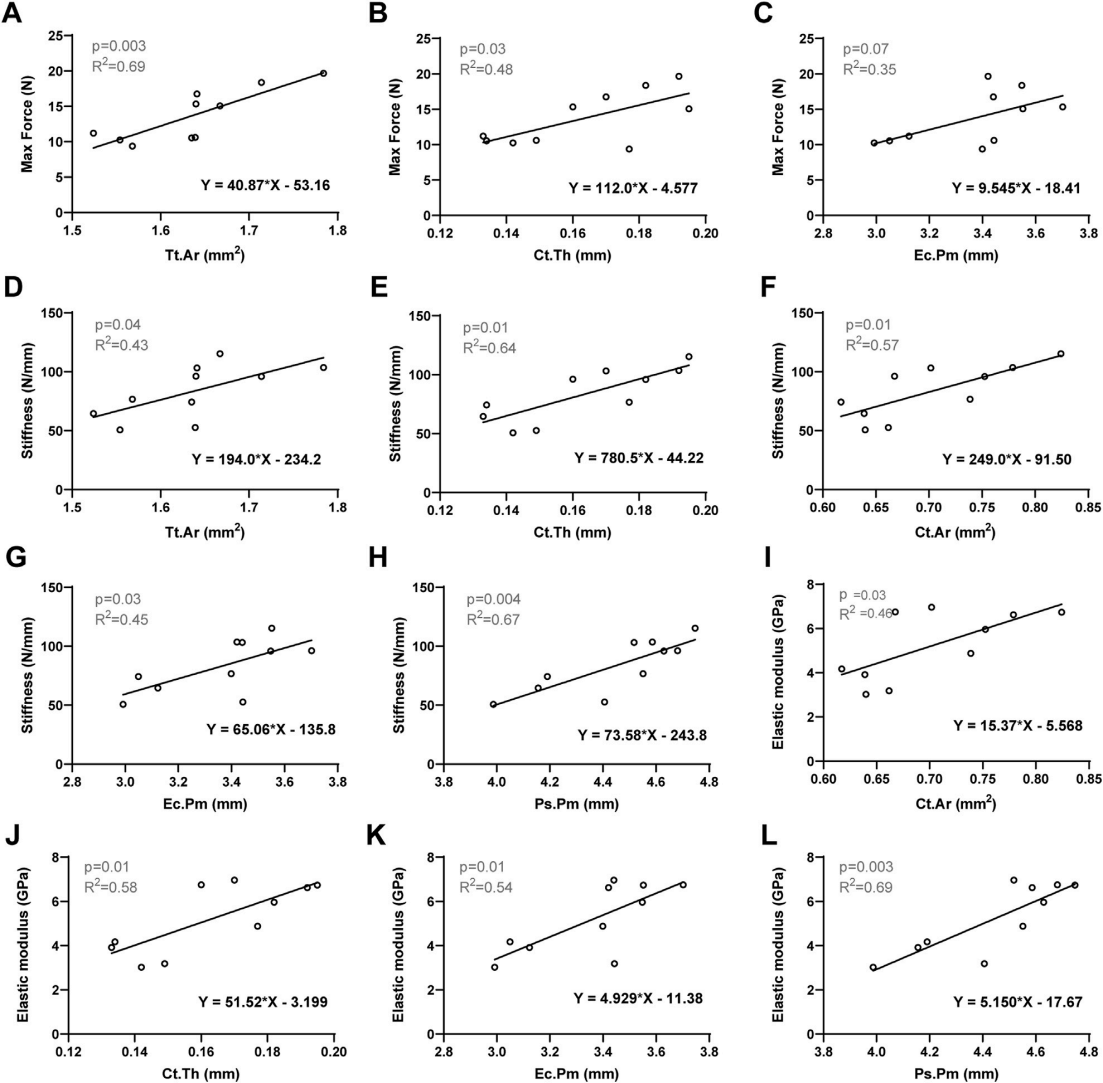
Linear regression analysis: The significant correlation between bone mechanical properties and the morphological parameters of cortical bone. **(A)** Max Force *versus* Tt.Ar. **(B)** Max Force *versus* Ct.Th. **(C)** Max Force *versus* Ec.Pm. **(D)** Stiffness *versus* Tt. Ar. **(E)** Stiffness *versus* Ct.Th. **(F)** Stiffness *versus* Ct. Ar. **(G)** Stiffness *versus* Ec.Pm. **(H)** Stiffness *versus* Ps.Pm. **(I)** Elastic modulus *versus* Ct. Ar. **(J)** Elastic modulus *versus* Ct.Th. **(K)** Elastic modulus *versus* Ec.Pm. **(L)** Elastic modulus *versus* Ps.Pm. Significant difference defined as *p* < 0.05.

To elucidate the biomechanical responses of femur observed in three-point bending tests, we investigated the correlations between bone mechanical properties and the morphological parameters of cortical bone. Specifically, we evaluated the associations between cortical bone area and/or cortical thickness with maximum force and stiffness. Pearson’s correlation analysis was conducted by consolidating all femurs into a single group, with a significance level set at 0.05. The significant Pearson correlation coefficients are presented in Table 2, while the correlations of interest are depicted (Figures 3A–L). Increasing cortical thickness and endocortical perimeter significantly enhanced the maximum force, stiffness, and elastic modulus, along with a positive and significant correlation between stiffness and both cortical cross-sectional area and periosteal perimeter.

**TABLE 2:** Correlation between femoral bone mechanical properties and cortical morphological parameters in 7-month-old female WT and Beclin-1^+/−^ mice.

*Pearson correlation coefficient*	Max force	Stiffness	Elastic modulus
Tt.Ar	0.831**	0.656*	0.622
Ct.Ar	0.605	0.755*	0.674*
Ct.Th	0.661*	0.806**	0.761*
Ec.Pm	0.639*	0.821**	0.833**
Ps.Pm	0.594	0.673*	0.738*

Significant difference: *p < 0.05, **p < 0.01.

### 3.3 Beclin-1 deficiency decreased tissue-level stress and strain in female murine femurs

**FIGURE 4:**
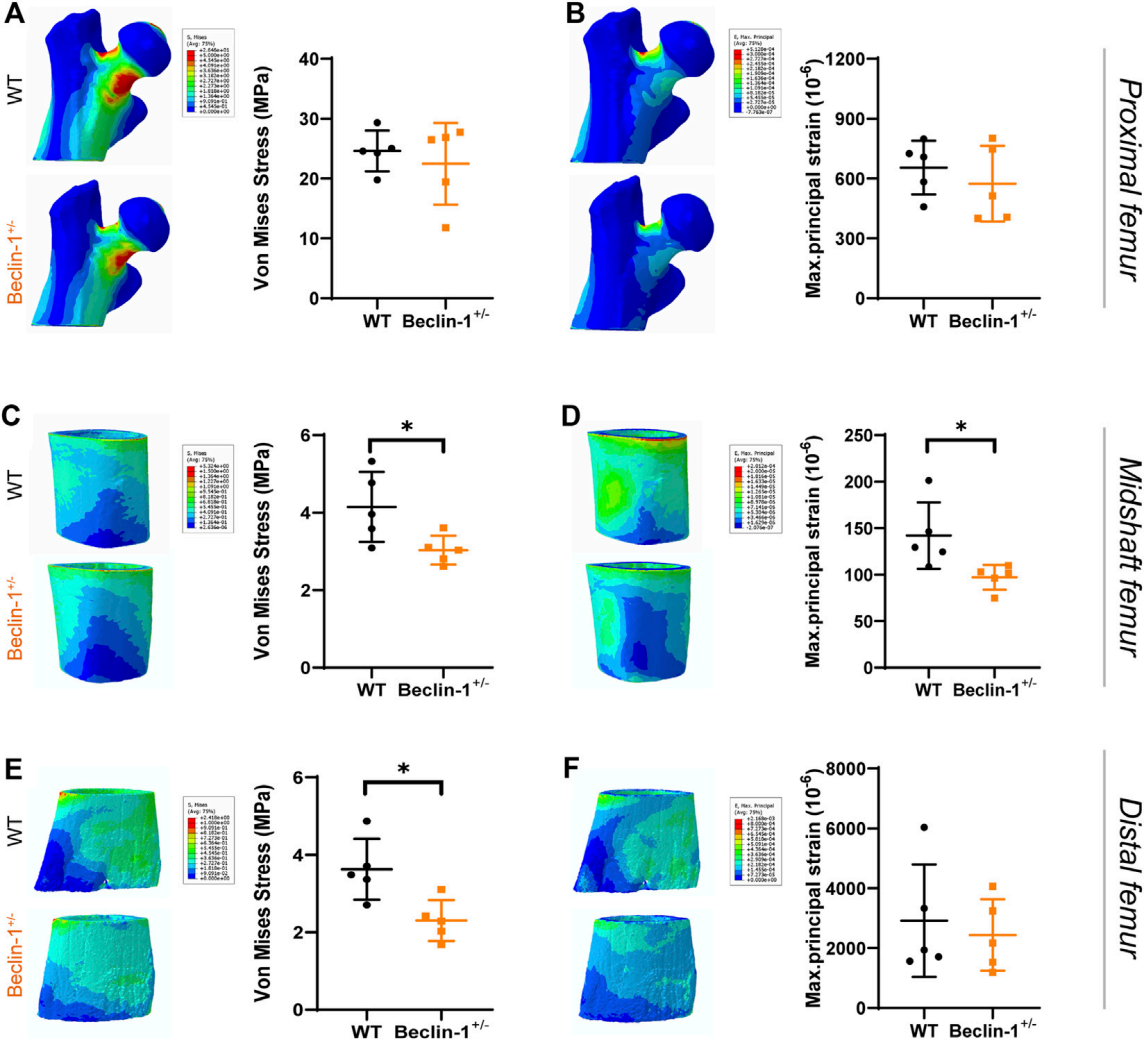
Predicted stress and strain distribution in femur of WT and Beclin-1^+/−^ female mice. **(A)** Von Mises stress and **(B)** Maximum principal strain of proximal femur. **(C)** Von Mises stress and **(D)** Maximum principal strain of midshaft femur. **(E)** Von Mises stress and **(F)** Maximum principal strain of distal femur. Significant difference: *p < 0.05.

FE models were established to simulate the peak physiological load on murine long bone and analyze the stress and strain of the bone tissue for the WT and Beclin-1^+/−^ mice. In the proximal region of femur, von Mises stress and maximum principal strain occurred at medial side of the femoral neck and top of femoral head, while these parameters were more evenly distributed throughout bone tissue in midshaft and distal regions (Figure 4). Under peak physiological load, the tissue-level stress and strain throughout the femurs were generally lower for the Beclin-1^+/−^ mice than for the WT mice. There were no significant differences in the stress or strain distributions between WT and Beclin-1^+/−^ mice in the femoral proximal region (Figures 4A, B). In femur midshaft, Beclin-1^+/−^ mice significantly reduced the distribution of von Mises stress and maximum principal strains (Figures 4C, D), while only von Mises stress significantly decreased in distal region (Figures 4E, F). Using FE models, the maximum principal strains on the surfaces of the midshaft femurs under the peak physiological load were predicted to be 142 ± 35.7 με for the WT mice and 97.1 ± 13.42 με for the Beclin-1^+/−^ mice, while the corresponding predicted maximum von Mises stresses were 4.15 ± 0.90 and 3.03 ± 0.37 MPa, respectively. In the distal regions, the von Mises stress for the Beclin-1^+/−^ mice (2.30 ± 0.53 MPa) was 36.64% lower than that for the WT mice (3.63 ± 0.79 MPa).

The FE models predicted lower SED values for Beclin-1^+/−^ mice compared to WT mice in the midshaft, and distal femur regions. The most significant difference (50% decrease) was observed in the midshaft region. According to stress intensity theory, an SF value less than one indicates a higher risk of fracture. Our FE modeling did not show significant differences in the SF for the proximal region between WT mice (5.83 ± 1.66) and Beclin-1^+/−^ mice (6.33 ± 2.66), despite a slight increment of SF for Beclin-1^+/−^ mice. For the midshaft and distal regions, the SF values for the Beclin-1^+/−^ mice (31.59 ± 4.09 and 27.59 ± 6.19) were 36.99% and 64.62% higher, respectively, than those of WT mice (23.06 ± 5.63 or 16.76 ± 3.35), indicating a greater resistance to fractures in the femoral diaphysis of Beclin-1^+/−^ mice compared to WT (Table 3).

**TABLE 3:** Predicted biomechanical indicators in cortical bone of 7-month-old female WT and Beclin-1^+/−^ femurs using FE model.

	WT	Beclin-1^+/−^	Diff (%)
***Proximal femur***			
Strain energy density	0.0219 ± 0.007	0.0297 ± 0.01	35.61
Min Safety factor	5.83 ± 1.66	6.33 ± 2.66	8.58
***Midshaft femur***			
Strain energy density	0.0006 ± 0.0003	0.0003 ± 0.0001*	−50
Min Safety factor	23.06 ± 5.63	31.59 ± 4.09*	36.99
***Distal femur***			
Strain energy density	0.014 ± 0.007	0.008 ± 0.002	−42.86
Min Safety factor	16.76 ± 3.35	27.59 ± 6.19*	64.62

Significant difference: *p < 0.05.

**FIGURE 5:**
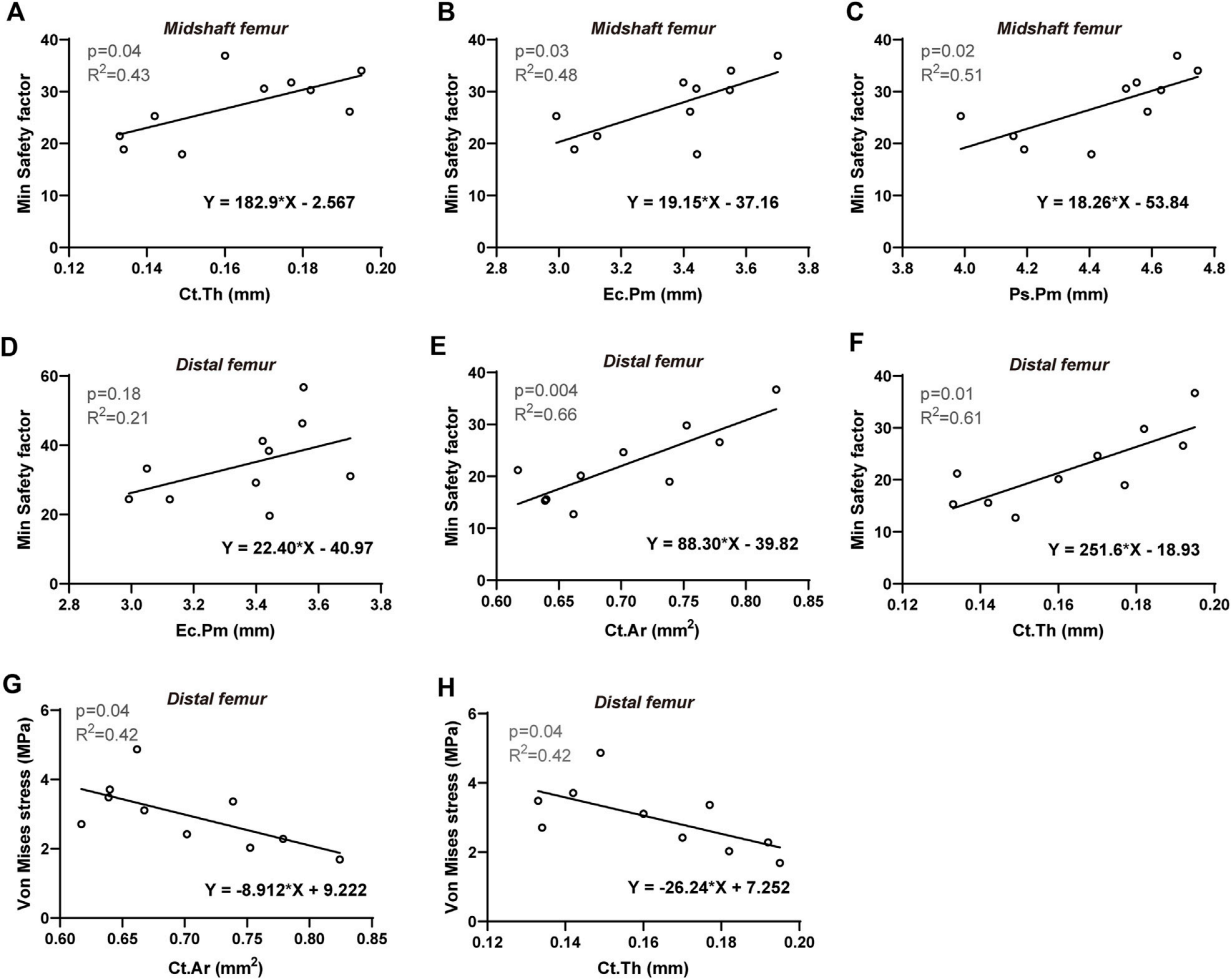
Linear regression analysis: The significant correlation between FE-predicted biomechanical indicators and morphological parameters of cortical bone. For midshaft femur: Min safety factor *versus* **(A)** Ct. Th, **(B)** Ec.Pm, and **(C)** Ps.Pm. For distal femur: Min safety factor *versus* **(D)** Ec.Pm, **(E)** Ct. Ar and **(F)** Ct. Th; Von Mises stress *versus* **(G)** Ct. Ar and **(H)** Ct. Th. Significant difference defined as *p* < 0.05.

**FIGURE 6:**
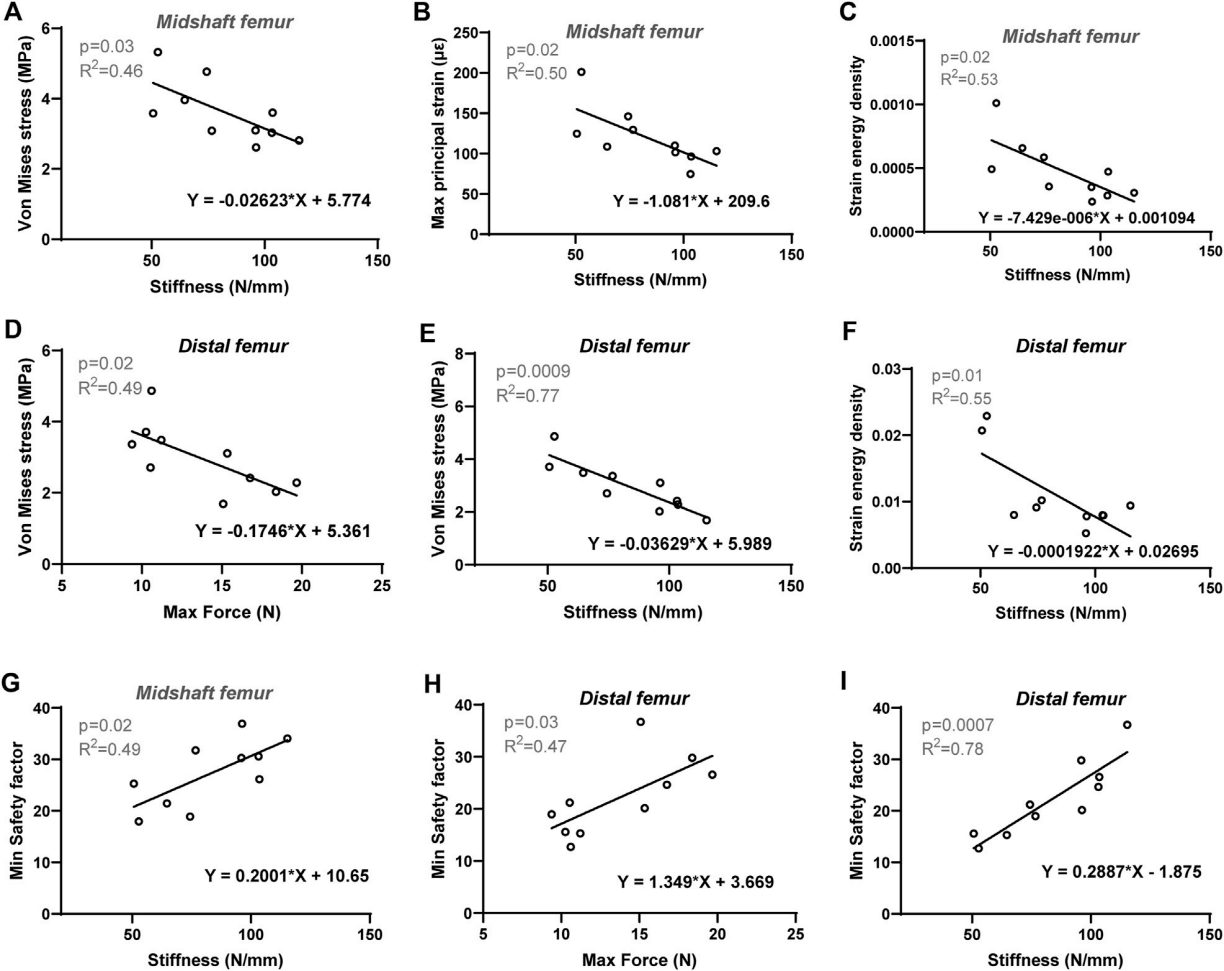
Linear regression analysis: The significant correlation between FE-predicted biomechanical indicators and experimental bone mechanical parameters of cortical bone. For midshaft region: Stiffness *versus* **(A)** Von Mises stress, **(B)** Max principal strain and **(C)** Strain energy density, **(G)** Min safety factor. For distal femur: Stiffness *versus* **(E)** Von Mises stress, **(F)** Strain energy density and **(I)** Min safety factor; maximum force *versus* **(D)** Von Mises stress and **(H)** Min safety factor. Significant difference defined as *p* < 0.05.

To further investigate the biomechanical indicators (von Mises stress, SF, maximum principal strain, and SED) of the femurs predicted by the FE model, their correlations with cortical bone morphological parameters as well as bone mechanical properties were evaluated, respectively. The Pearson correlation coefficients are summarized in Table 4. For the proximal femur, neither morphological nor mechanical parameters had an impact on the biomechanical indicators predicted by FE. However, increases in cortical thickness and perimeter significantly enhanced the SF values for both the midshaft and distal regions, accompanied by a positive and significant correlation between SF and cortical cross-sectional area in the distal femur (Figures 5A–F). Furthermore, von Mises stress in the distal region is negatively correlated with cortical bone thickness and area (Figures 5G, H). Considering its correlation with bone material properties on the midshaft femur, von Mises stress, maximum principal strain, and SED were negatively correlated with the stiffness and elastic modulus (Table 4; Figures 6A–C). Furthermore, maximum force, stiffness, and elastic modulus exhibited negative correlations with von Mises stress in the distal region (Figures 6D–F). Moreover, SF increased for both distal and midshaft femur when elevated bone mechanical properties were present particularly stiffness (Figures 6G–I).

**TABLE 4:** Correlation of FE-predicted biomechanical indicators with experimental bone morphological and mechanical parameters in 7-month-old female WT and Beclin-1^+/−^ femurs.

*Pearson correlation coefficient*	Bone morphology					Bone mechanical properties		
	Tt.Ar	Ct.Ar	Ct.Th	Ec.Pm	Ps.Pm	Max force	Stiffness	Elastic modulus
***Proximal femur***								
Von Mises stress	−0.158	−0.545	−0.453	−0.113	0.084	−0.134	−0.362	−0.209
Max principal strain	−0.298	−0.203	−0.195	−0.014	0.058	−0.215	−0.251	−0.192
Strain energy density	0.471	0.63	0.591	0.559	0.437	0.518	0.498	0.439
Min Safety factor	−0.183	−0.032	−0.05	0.011	0.013	0.173	0.131	0.151
***Midshaft femur***								
Von Mises stress	−0.116	−0.547	−0.614	−0.561	−0.512	−0.486	−0.680*	−0.733*
Max principal strain	−0.194	−0.394	−0.436	−0.323	−0.226	−0.631	−0.706*	−0.745*
Strain energy density	−0.185	−0.465	−0.554	−0.505	−0.403	−0.485	−0.727*	−0.761*
Min Safety factor	0.185	0.575	0.669*	0.715*	0.695*	0.456	0.703*	0.765**
***Distal femur***								
Von Mises stress	−0.549	−0.652*	−0.653*	−0.521	−0.304	−0.700*	−0.875**	−0.782**
Max principal strain	−0.132	−0.424	−0.349	−0.437	−0.251	−0.144	−0.456	−0.346
Strain energy density	−0.331	−0.376	−0.39	−0.491	−0.34	−0.577	−0.740*	−0.728*
Min Safety factor	0.629	0.822**	0.809**	0.677*	0.477	0.688*	0.886**	0.767**

Significant difference: *p < 0.05, **p < 0.01.

## 4 Discussion

The present study aimed to characterize the morphological and biomechanical behavior of femurs from adult female Beclin-1^+/−^ mice. Compared to WT mice, Beclin-1^+/−^ mice exhibited increased cortical bone area, perimeters and cortical thickness. Biomechanical assessment using three-point bending revealed that Beclin-1^+/−^ mice showed elevated fracture resistance with greater ultimate force and stiffness in the femoral diaphysis. These findings suggest that Beclin-1 deficiency improves the mechanical behavior of cortical bone, primarily due to thickening of the cortex. Additionally, FE analysis predicted reduced tissue-level stress and strain within the femurs of Beclin-1^+/−^ mice compared to those of WT mice under peak physiological load for long bones. In general, the stress and strain distributions were similar among each femoral model, showing that the highest von Mises stress and maximum principal strain occurred at the medial side of the femoral neck in the proximal region and were more uniformly distributed throughout the bone tissue, with a larger difference in the midshaft femur. The SED values for the midshaft femurs from the Beclin-1^+/−^ mice tended to be lower than those observed for the WT mice. SF values for the midshaft and distal regions were significantly higher for Beclin-1^+/−^ mice than for WT mice, indicating that female Beclin-1^+/−^ mice cortical bone exhibited better fracture resistance.

In this study, Beclin-1 deficiency in 7-month-old female mice led to an increase in cortical bone mass, but a slight decrease in trabecular bone mass and lower BMD. These reciprocal effects are intriguing, as they match trends observed across different mouse strains. For instance, strains with low bone density, such as C57BL/6J (B6), generally have thin cortices but extensive trabecular bone; while other strains, such as C3H/HeJ (C3H), exhibit much thicker cortical bone but less trabecular bone extending from the growth plate, compared to B6 mice (Turner et al., 2000). Cortical bone loss resulted from impaired periosteal bone formation and increased endocortical bone resorption. Trabecular bone loss was caused by reduced trabecular bone formation and increased bone resorption (Funck-Brentano et al., 2018). The trabecular osteopenia caused by Beclin-1 deficiency is interesting in that both trabecular thickness and separation are decreased, and trabecular number density increases. This is unlike, for instance ovariectomy, where separation is increased and number falls (Syed et al., 2010; He et al., 2011); it suggests a changed pattern of trabecular turnover and architecture, toward a more finely textured pattern.

The size and thickness of cortical bone are key determinants of bone strength and fracture risk (Poole et al., 2010). Increasing age was also found to be related to a decrease in periosteal attachment and an increase in intracortical resorption, thus weakening bone material and structural integrity (Seeman, 2008b). The increased cortical area and/or thickness observed in Beclin-1^+/−^ female mice is likely due to enhanced periosteal apposition on the periosteal envelope. Periosteal expansion at the diaphysis involves bone formation on the periosteal surface to increase bone width (Isojima and Sims, 2021). During skeletal growth, periosteal apposition surpasses endosteal resorption, leading to a net increase in bone width and cortical thickness, crucial for the size and shape of developing bones (Chen et al., 2022). Following the cessation of longitudinal growth, a balanced dynamic between periosteal apposition and endocortical formation is essential to maintain cortical thickness, bone diameter, and strength. However, an imbalance between periosteal apposition and endocortical formation, with less new bone formation, increased osteoclast activity, and more endocortical bone loss, can lead to thinner cortical bones, greater fragility, and defects in fracture repair and bone regeneration (Brommage et al., 2019; Chen et al., 2019; Salazar et al., 2019; Isojima and Sims, 2021). Bone loss from the endocortical surface contributes to bone fragility, while bone deposition on the periosteal surface might be an adaptive response to maintain resistance to bending (Szulc et al., 2006). The larger cross-sectional area of the Beclin-1^+/−^ mice may be due to a stronger adaptive response to mechanical loading. Cortical anabolic responses to mechanical stimuli declined with age into adulthood and cortical cross-sectional geometry alone does not necessarily predict whole-bone functional stiffness (Main et al., 2014). Millard et al. reported that the increase in biomechanical strength of the femurs in aging female mice may be attributable to an increase in bone formation by endothelial osteoblasts (Millard et al., 2017). Tissue material properties and mechanical stress both contribute to bone strength, and changes in the material properties may offset the increase in bone volume (Donnelly, 2011). The skeletal phenotypic features observed in female femurs, increased cortical thickness, and reduced tissue-level stress and strain, are in agreement with previous observations (Endo et al., 2020) illustrating that reduced cortical bone thickness caused strong stresses and strains in female femoral shafts and consequently increased the risk of hip fracture.

The material composition and structural characteristics of bone jointly affect bone strength, where the structure determines the load that can be tolerated and the load also determines the structure, and bone modifies its material composition and structure by adaptive modeling and remodeling to accommodate loads (Seeman, 2008a). The cortical bone structural properties, including cortical thickness, cross-sectional area, and area moment of inertia have been known to provide mechanical competence and can be applied to predict bone strength and fracture risk (Augat and Schorlemmer, 2006). Although size and shape are important morphological characteristics that determine bone strength, the composition and mechanical properties of bones vary as a function of age (Mumtaz et al., 2020). The bone mineral content increasing with age was previously shown to lead to increased maximum force, higher elastic modulus, lower work-to-fracture, and ultimately reduced bone toughness (Currey, 1984). Further investigations are necessary to understand the cortical anabolic response to mechanical loading in the altered bone microstructure of female Beclin-1^+/−^ mice.

There were several limitations in the present study. First, our assessment is largely associated with the accuracy of the simulation and the correct description of the boundary conditions. Differences in bone morphology and the loading distribution on cortical bone may compromise the accuracy of non-invasive bone strength assessments. (Arrington et al., 2006). Individual variations in tissue properties such as porosity and mineralization impact bone strength. The FE model did not account for changes in intracortical porosity due to the resolution of the microCT images, roughly matching the average vascular aperture of 9–15 μm, potentially lowering the prediction accuracy (Varga et al., 2020). Furthermore, the increased brittleness caused by increased mineralization is associated with the bone mineral density distribution (Roschger et al., 2008). Since there were no significant differences in bone strength on the tissue scale for the proximal femur, these results cannot be directly compared with the previous literature. Although there is strong evidence that the deterioration of biomechanical properties at the whole bone level is the cause of bone brittleness, the current study has not addressed the cellular and molecular basis of the actual changes in osteogenic mechanical properties. Future work focused on understanding the underlying collagen structure and how it can change could provide a means of addressing this question. Lastly, in the morphometric and FE analyses, we displayed heat maps for specific regions rather than the entire bone. Although previous studies have focused on measuring cortical bone in the midshaft region, this approach may not always be optimal, especially for morphological analysis using Micro-CT 3D imaging. The midshaft may represent the weakest point of the long bone, showing minimal response, whereas larger responses might be observed in other areas, such as the metaphyseal region (Javaheri et al., 2015; Javaheri et al., 2018). Imaging the entire length of murine bones could provide a more comprehensive view of architectural changes and mechanical performance (Javaheri et al., 2020). Further investigation is crucial to fully understand the changes in bone microstructure and the distribution of stress and strain at the tissue level throughout the whole bone in female Beclin-1^+/−^ mice.

In conclusion, we observed striking alterations in the morphological and biomechanical properties of femurs from adult female Beclin-1^+/−^ mice, providing valuable information on bone strength and fracture prediction, thus potentially contributing to the treatment and prevention of osteoporosis and other bone diseases. Further integration of experimental and computational research is necessary to elucidate the underlying mechanisms responsible for increased bone strength in female Beclin-1^+/−^ mice, as well as to investigate the cellular and molecular basis of the cortical anabolic response to mechanical loading in altered bone microstructure.

## Data Availability

The original contributions presented in the study are included in the article, further inquiries can be directed to the corresponding authors.
